# Differences in childhood body mass index between lesbian/gay and bisexual and heterosexual female adolescents: A follow-back study

**DOI:** 10.1371/journal.pone.0196327

**Published:** 2018-06-18

**Authors:** Kate Keenan, Kristen Wroblewski, Phoenix A. Matthews, Alison E. Hipwell, Stephanie D. Stepp

**Affiliations:** 1 Department of Psychiatry and Behavioral Neuroscience, University of Chicago, Chicago, Illinois, United States of America; 2 Department of Public Health Sciences, University of Chicago, Chicago, Illinois, United States of America; 3 College of Nursing, University of Illinois at Chicago, Chicago, Illinois, United States of America; 4 Department of Psychiatry, University of Pittsburgh, Pittsburgh, Pennsylvania, United States of America; McMaster University, CANADA

## Abstract

**Objective:**

To determine whether childhood body mass index (BMI), assessed in childhood, differs between lesbian/gay and bisexual (LGB) and heterosexual late adolescents, and whether childhood social stressors impact the association between sexual orientation and childhood BMI.

**Methods:**

Participants included 2,070 late adolescents from the Pittsburgh Girls Study, of whom 233 (11.2%) identified as lesbian or bisexual and 1,837 (88.8%) as heterosexual at ages 17–20 years. Weight and height were used to calculate body mass index (BMI) at ages 10 through 14 years. Data were collected on child reported loneliness at ages 8 to 10 and peer victimization from 10 to 14 years.

**Results:**

LGB females had higher BMIs and greater increases in BMI from ages 10–14 years compared to heterosexual females and reported higher levels of loneliness and peer victimization in childhood. Loneliness moderated the association between sexual identity and changes in BMI; for participants with loneliness scores in the upper quartile, the increase in BMI over time was approximately 30% higher for LGB females compared to heterosexual females. Child report of peer victimization mediated the association between sexual identity and changes in BMI, with nearly 18% of the total effect of sexual identity on BMI over time accounted for by peer victimization.

**Conclusions:**

Lesbian and bisexual adolescents report greater loneliness and peer victimization as children than heterosexual adolescents; these stressors confer risk for higher BMI among LGB females. These data underscore the importance of research on the social determinants of health. The hypothesis that the social stressors may partially account for differences in BMI and other cardiometabolic risk factors between LGB and heterosexual females should be addressed in future research.

## Introduction

Data from several large surveys provide evidence of significant health disparities for sexual minority females. Obesity and asthma are reported more frequently among lesbian/gay and bisexual (LGB) women than among heterosexual women;[1] and bisexual women were more likely to be at increased risk for diabetes and hypertension compared to heterosexual women.[2] LGB women also report poorer physical health and greater disability,[3] including early diagnosis of heart disease.[4] This is in contrast to sexual minority men, who are less likely to be obese and tend not to differ from heterosexual males in terms of cardiometabolic disease.[5] Against this backdrop of emerging data indicating health disparities among sexual minorities, the National Institutes of Health requested the Institute of Medicine (IOM) to assess the current state of knowledge of the health of lesbian, gay, bisexual, and transgender people. The committee’s conclusion, published in 2011, was that the state of knowledge as to how sexual identity and gender identity influence health was sparse; one area of research cited in the report as particularly underdeveloped was the health status of sexual minority youth.[6]

In the present longitudinal study, we begin to address this gap by examining whether reported differences in health indices between LGB and heterosexual females emerge earlier in development by focusing on differences in childhood BMI. Childhood BMI is prospectively associated with obesity and atherosclerosis.[7–9] Determining the age at which differences in BMI emerge is important for testing causal models and for developing preventive interventions. Self-reported data on height and weight from the US National Longitudinal Study of Adolescent Health, showed that non-Latina white and Latina white bisexual females had higher BMIs than non-Latina white and Latina white heterosexuals, respectively in adolescence and adulthood.[10] BMI based on self-report was higher among sexual minority adolescent girls compared to heterosexual girls in the Growing Up Today Study (GUTS), which comprises offspring of the Nurses’ Health Study II.[11] Among high school students in Massachusetts, bisexual and lesbian/gay girls were more likely to be overweight or obese based on self-reported height and weight, then heterosexual girls.[12]

A second goal of the present study is to incorporate hypotheses related to the social determinants of health; the impact of social factors on health[13] including the experience of discrimination, social isolation, and victimization.[14–16] Much of the research in this area has been conducted in adults, but there is evidence that social factors also impact the health of children and adolescents. As early as adolescence, for example, discrimination and unfair treatment as a result of minority status (e.g., race, poverty) are associated with cardiometabolic and neuroendocrine health risks.[17,18] Peer victimization in 5^th^ grade was prospectively associated with physical health in 10^th^ grade.[19] Compared to males, the health of females may be particularly impacted by social stressors: in one study recall of childhood stressors was associated with increases in BMI over time for females, but not for males.[20] Data from the Youth Risk Behavior Survey, demonstrated greater odds of peer victimization for overweight sexual minority females compared to overweight heterosexual females.[21]. Thus, we test whether adolescents who identify as a sexual minority experienced higher levels of social stressors as children than adolescents who identify as heterosexual, whether the association between sexual minority status and BMI varied by social stress exposure.

These hypotheses are tested using data from a longitudinal study that began in childhood. We use a follow-back approach in that we identify participants as LGB or heterosexual in late adolescence, and then use prospectively collected data on child report of social stressors and interviewer collected measures of height and weight to test associations with sexual identity. Although the follow-back approach allows data on social stressors to be assessed independent of the assessment of sexual identity, the underlying assumption is that females who identify as a sexual minority in late adolescence are likely to experience same-sex attraction in childhood, which may lead to feelings of isolation and/or victimization.

## Methods

The Pittsburgh Girls Study (PGS) includes a representative sample of girls from the City of Pittsburgh, Pennsylvannia.[22] A stratified, random household sampling, with over-sampling of households in low-income neighborhoods, was used to identify girls who were between the ages of 5 and 8 years. Neighborhoods in which at least 25% of the families were living at or below the poverty level were fully enumerated, and a random selection of 50% of the households in all other neighborhoods was enumerated during 1998 and 1999. Weights were calculated to account for the oversampling of low-income neighborhoods based on U.S. census data. In the present study, all analyses are conducted using sampling weights.

The enumeration identified 3,118 separate households in which an eligible girl resided. From these households, families who were moving out of state and families in which the girl would be age ineligible by the start of the study were excluded. Of the 2,992 eligible families, 2,875 (96%) were successfully re-contacted to determine their willingness to participate in the study. Of those families, 85% agreed to participate, resulting in a sample size of 2,450. In-home interviews were conducted annually beginning in 2000 when the girls were between the ages of 5–8 years. The University of Pittsburgh Institutional Review Board approved all study procedures. Written informed consent was obtained from the primary caregiver, and from the girls when they reached the age of 18 years. Trained interviewers conducted separate, private interviews with the caregivers and adolescents. Participation rates have been at or above 85% in all assessment years. In the present study, data on sexual identity were derived from the 13^th^ annual assessment wave, when girls were 17–20 years of age. We then compare sexual identity groups (lesbian/bisexual or heterosexual) on changes BMI from ages 10–14 years, peer victimization from 10–14 years, and Loneliness from ages 8–10 years.

Of the 2,450 PGS participants, 2,079 (85%) were interviewed at wave 13 during late adolescence, when they were 17–20 years of age. A single question was administered to assess sexual identity: *Do you consider yourself to be*: *Heterosexual or straight*, *Gay or lesbian*, *or Bisexual*. Five participants reported that they didn’t know how to identify their sexual identity and 4 refused to answer the question. These 9 participants (0.4%) were excluded from the analyses. Of the remaining 2,070 participants, 233 (11.2%) identified as lesbian or bisexual and 1,837 (88.8%) as heterosexual. These rates are slightly higher but comparable to those reported in the National Survey of Family Growth.[23]

Among the LGB participants in PGS, 52% identified as Black American, which was not significantly different from the heterosexual participants (46%) (*Chi-square* [1] = 3.351, *p* = .067). As has been reported previously,[22] participants not interviewed (those who refused to participate or were not reachable) were more likely to be White American (*Chi-square* [1] = 29.90, *p* < .001).

Interviewers recorded the participants’ heights and weights during home visits, using a calibrated scale and stadiometer, which were used to calculate absolute BMI (weight (kg)/[height (m)]^2^) from ages 10–14 years.

Victimization by peers was measured by self-report at ages 10–14 years using *The Peer Victimization Scale* (PVS).[24] This nine-item scale includes victimization by physical aggression and exclusion rated on a scale ranging from never (0) to a few times per week (4). Internal consistency was high with *alpha* coefficients calculated at each age ranging from .74 to .89. The modal score on the PVS in each year was 0 and the median was 2, with the exception of age 14 for which the median was 1. Thus, most participants reported scores of 2 or lower in each year.

The *Loneliness and Social Dissatisfaction Scale* (LSDQ) was administered to the girls at ages 8 through 10 years: the upper age for which the scale was validated.[25] Total scores were calculated for 16 items measuring social relations in four domains: feelings of loneliness (e.g., *Are you lonely at school*?), perceptions of peer relations (e.g., *Do you have lots of friends at school*?), appraisals of whether important relationship provisions are being met (e.g., *Are there kids you can go to when you need help*?), and feelings of social competence (e.g., *Are you good at working with other kids at school*?). Response choices for individual items ranged from 0 to 2; positive items were reversed coded such that a higher score indicated higher levels loneliness and social dissatisfaction; *alpha* coefficients for the total scores at each age and ranged from .88 to .91. The modal score on the LSDQ in each year was 0 and the median scores were 3, 2, and 1 at ages 8, 9, and 10 years, respectively.

*Attrition*. There were no differences in BMI or changes in BMI per year between those interviewed and those not interviewed in wave 13. Individuals not interviewed in wave 13 reported *lower* levels of peer victimization at age 10 than those interviewed (*mean* = 3.74 versus 4.73, *p* = .011) and *lower* levels of loneliness at age 8 (*mean* = 4.33 versus 5.06, *p* = .046) but no difference in changes in peer victimization and loneliness over time. We examined sexual orientation in the two previous waves (i.e., waves 11 and 12) as a function of those not interviewed in wave 13 and found no difference in the sexual orientation of those girls who were interviewed and those who were not interviewed in wave 13.

### Statistical analyses

To characterize change in BMI, and youth report of loneliness and peer victimization, individual slopes were extracted for each participant for each of the three repeatedly measured study variables: BMI, loneliness, and peer victimization (e.g., for BMI, a slope was calculated for each individual and this summary measure of change was used as the dependent variable in a linear regression model). This approach was appropriate given the dependence of the repeated measures, and the different number and ages of assessments for the three variables.[26] For BMI and peer victimization, this reflected the change from ages 10–14 years; for loneliness the individual slopes were based on changes in scores from ages 8 to 10 years. Differences in the individual slope coefficients, in addition to the observed baselines values, were tested as a function of sexual identity. Given known race differences in BMI [27] we included race as a covariate in all analyses.

For tests of moderation, hypothesized correlates were entered in four steps in a single model: 1) BMI at age 10 and race; 2) sexual identity; 3) loneliness and peer victimization; and 4) the interaction of loneliness and peer victimization with sexual identity. All analyses were conducted using IBM SPSS Statistics, Version 22 with weighted data.

We tested mediation effects of loneliness and peer victimization on changes in BMI over time in stepwise regression models: step 1) BMI at age 10 and race; step 2) sexual identity; step 3) loneliness or peer victimization. Mediation effects were calculated using a regression-based bootstrapping approach with k = 5,000 re-samples and 95% bias corrected confidence intervals using the PROCESS macro in SPSS.[28] This approach is a powerful method for estimating indirect effects, even in large samples, in part because it does not rely on assumptions of normality.[29] A significant mediation effect was considered to have occurred if the 95% confidence interval for the indirect effect did not contain zero. Effect sizes were calculated from percent mediation.

## Results

Descriptive statistics and comparisons for BMI, and self-reports of loneliness and peer victimization between heterosexual and LGB participants are presented in Table 1. Lesbian/gay and bisexual females did not differ from each other in BMI at age 10 (mean = 21.6 versus 22.0), or in loneliness (mean = 6.5 versus 6.2) or peer victimization (mean = 6.2 versus 7.0) at age 8 or in change in BMI, loneliness, or peer victimization over time, thus the two groups were combined into a single group comprising LGB females.

**Table 1:** Descriptive statistics and comparisons of BMI, loneliness and peer victimization for heterosexual and LGB participants.

	Total Sample		Heterosexual		LGB		Heterosexual vs LGB	
	Mean	SD	Mean	SD	Mean	SD	*p* value	*d*
Body mass index (BMI) at age 10	20.2	4.9	20.0	4.7	21.9	5.7	< .001	.22
BMI changes per year ages 10–14	1.0	0.7	1.0	0.7	1.2	0.9	< .001	.15
Loneliness at age 8	4.9	5.8	4.8	5.7	6.3	6.6	.001	.15
Loneliness changes per year ages 8–10	-0.7	3.2	-0.7	3.1	-0.4	4.0	.126	.06
Peer victimization at age 10	4.5	5.9	4.5	5.6	6.8	7.1	< .001	.25
Peer victimization changes per year ages 10–14	-0.6	1.5	-0.5	1.4	-0.7	1.9	.175	.06

Note: absolute BMI is calculated as: (weight (kg)/[height (m)]^2^); All tests of mean differences controlled for race

**Fig 1:**
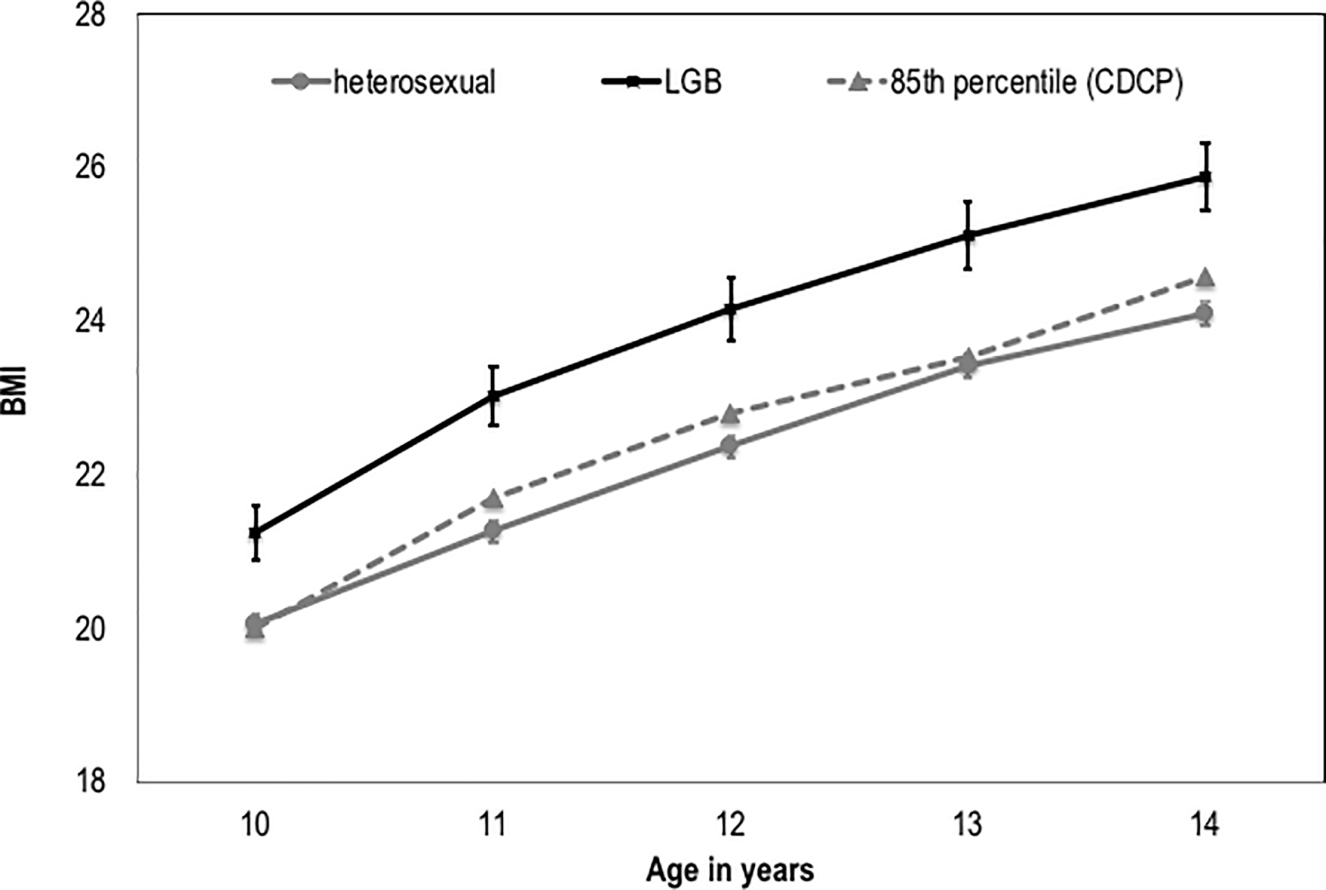
Change in body mass index by sexual identity. BMI = Body Mass Index; heterosexual = heterosexual participants; LGB = lesbian/gay or bisexual participants; CDCP = Center for Disease Control and Prevention. Group effect of sexual identity on BMI: *F* [1, 1714] = 17.462, *p* < .001, *cohen’s d* = .67; Interaction effect of sexual identity by time on BMI: *F* [2.64, 4517.89] = 4.126, *p* < .01, *cohen’s d* = .09. Data are estimated means standard errors at each time point within group. The 85^th^ percentile is indicated for each age according to data provided by the Centers for Disease Control and Prevention.

On average, LGB females had higher BMIs at age 10 (mean = 21.9 versus 20.0, *cohen’s d* = .22) and greater increases in BMI from ages 10 to 14 (mean change per year = 1.2 versus 1.0, *cohen’s d* = .15) than heterosexual participants. According to age- and sex-specific Centers for Disease Control and Prevention normative data, children and adolescents whose body mass indices fall at or above the 85^th^ percentile are considered overweight or obese.[30] Thus, we include the 85^th^ percentile as a reference point in Fig 1, along with the estimated means for the heterosexual and LGB participants. Beginning at age 10 years, the average BMI for LGB participants was above the 85^th^ percentile for age and remained above at each age. In contrast, the average BMI for the heterosexual participants fell at or just below the 85^th^ percentile at each age (Fig 1).

Compared to heterosexual participants, LGB participants reported higher levels of loneliness at age 8 (mean = 6.3 versus 4.8, *cohen’s d* = .15) and higher levels of peer victimization at age 10 (mean = 6.8 versus 4.5, *cohen’s d* = .25) (Table 1).

A multivariate linear regression was computed to assess the relative contribution of BMI at age 10, sexual identity and peer social stressors, and their interactive effects on changes in BMI from ages 10 to 14 years in a single model. As shown in Table 2, BMI at age 10 (*beta* = .282, p < .001), race (*beta* = .096, *p* < .001), and the interaction of loneliness at 8 and sexual identity (*beta* = .053, *p* < .05), were significantly associated with change in BMI over time. The interaction effect between peer victimization at age 10 and sexual identity was slightly smaller in absolute magnitude compared to the loneliness and sexual identity interaction but was not statistically significant (*p* = .562).

**Table 2:** Test of moderation of social stressors on the association between sexual identity and change in BMI from ages 10–14 years.

Dependent Measure: change in BMI from 10–14 years	Standardized Coefficients β	t value	p level
BMI (age 10)	**.282**	**12.847**	**< .001**
Black American vs. White American	**.096**	**4.391**	**< .001**
LGB vs. heterosexual (sexual identity)	.033	1.437	.151
Loneliness at age 8 (centered at the mean)	.000	0.016	.987
Peer victimization at age 10 (centered at the mean)	.108	1.457	.145
Sexual identity x loneliness at age 8	**.053**	**2.163**	**.036**
Sexual identity x peer victimization at age 10	-.043	-0.580	.562
	*F*	*df*	*adj R*^*2*^
Overall Model Statistics	**35.09**	**7,1920**	**< .001**

Note: absolute BMI is calculated as: (weight (kg)/[height (m)]^2^); Bolded parameters are statistically significant. All main and interaction effects tested within the same model, controlling for race.

**Fig 2:**
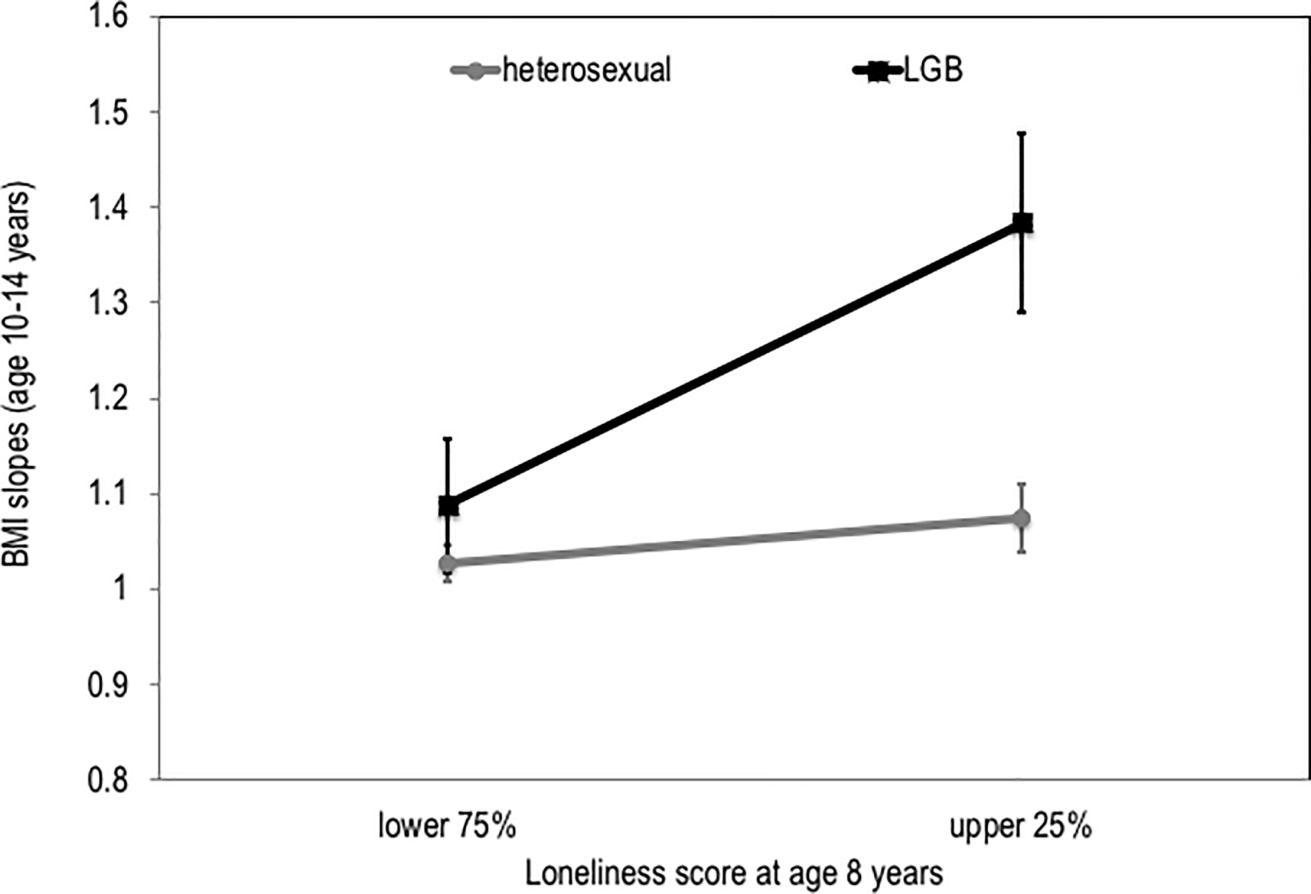
Moderation effect of loneliness on the association between sexual identity and changes in BMI from ages 10–14 years. BMI = Body Mass Index; heterosexual = heterosexual participants; LGB = lesbian/gay or bisexual participants. Average slopes for BMI from ages 10 to 14 years for LGB participants and heterosexual participants at low and high levels of self-reported loneliness at age 8 years. Controlling for race, BMI and peer victimization at age 10, the slope for heterosexual participants did not differ as a function of high loneliness (*F* [4, 1660] = 1.443, *p* = .230, *cohen’s d* = .06), whereas the slope for LGB participants did differ (*F* [4, 209] = 6.124, *p* = .014, *cohen’s d* = .34).

The significant interaction effect between loneliness at age 8 and sexual identify on changes in BMI from 10 to 14 years was further probed by graphing the average slope for BMI for heterosexual and LGBN participants whose scores on the loneliness scale at age 8 years fell in the upper 25% of the distribution for the sample or the lower 75% (Fig 2). Controlling for race, BMI and peer victimization at age 10, the slope for heterosexual participants did not differ as a function of high loneliness (*F* [4, 1660] = 1.443, *p* = .230, *cohen’s d* = .06), whereas the slope for LGB participants did differ (*F* [4, 209] = 6.124, *p* = .014, *cohen’s d* = .34).

The mediation analyses revealed significant effects of peer victimization at age 10 but not of loneliness on changes in BMI over time. As shown in Table 3, the effect of sexual minority status on change in BMI was reduced when peer victimization was added to the model (, after controlling for age 10 BMI and race: the standardized beta was reduced from .046 to .038. The indirect effect estimate was 0.008 (95% confidence interval = 0.001–0.016), yielding an effect ratio of 0.179. The percent of the total effect on change in BMI from ages 10–14 attributed to the indirect effect of peer victimization at age 10 was 17.9%.

**Table 3:** Test of mediation of peer victimization on the association between sexual identity and change in BMI from ages 10–14 years.

Dependent Measure: change in BMI from 10–14 years	Standardized Coefficients β	t value	p level
Step 1			
BMI (age 10)	**.292**	**13.487**	**< .001**
Race	**.102**	**4.728**	**< .001**
Step 2:			
BMI (age 10)	**.287**	**13.188**	**.000**
Race	**.102**	**4.701**	**.000**
Sexual identity	**.046**	**2.129**	**.033**
Step 3			
BMI (age 10)	**.284**	**13.060**	**.000**
Race	**.095**	**4.364**	**.000**
Sexual identity	.038	1.731	.084
Peer victimization at age 10 (centered at the mean)	**.073**	**3.364**	**.001**
	F	df	p level
Overall Model Statistics	**60.498**	**4, 1939**	**< .001**

Note: absolute BMI is calculated as: (weight (kg)/[height (m)]^2^); Bolded parameters are statistically significant.

## Discussion

The results of the present study extend the existing literature on health disparities among LGB females by providing evidence that a health risk associated with significant morbidity differs as a function of sexual identity in a representative, community-based sample of adolescents. Compared to heterosexual participants, LGB participants had higher body mass indices during early to middle adolescence. A higher BMI has been reported among adult LGB women.[31,32] Prospective data from Nurses’ Health Study II demonstrated continued weight gain for lesbian and bisexual women relative to heterosexual women from their mid-20s through age 59 years.[33] BMI based on self-report was higher among sexual minority adolescent girls compared to heterosexual girls in the GUTS study, which comprises offspring of the Nurses’ Health Study II.[11] In the GUTS study, data on BMI in early adolescence (ages 12–14) were available from a relatively small number of repeated observations from lesbian and bisexual adolescents (total number of observations = 68).[11] The present study provided an opportunity to extend the results from the existing literature by starting earlier in development and using objective measurements of height and weight collected by trained interviewers, resulting in one of the first sufficiently powered reports on childhood BMI among females who later identify as lesbian or bisexual.

The observed differences in BMI in childhood as a function of sexual identity suggests that one pathway to later health disparities may be via contextual experiences that are developmentally salient for children. Based on strong findings in the adult literature,[14–16] and emerging results in studies of children and adolescents,[17–19] the social context was the focus of the present study. In addition to BMI, youth report of loneliness and peer victimization in childhood differed for LGB and heterosexual females. LGB participants reported higher rates of childhood loneliness and victimization and both of these social stressors differentially conferred risk for increases in BMI for LGB adolescents. The increase in BMI over time among those with high levels of childhood loneliness was approximately 30% higher for LGB participants compared to heterosexual participants, and peer victimization accounted for close to 18% of the association between sexual minority status and changes in BMI from ages 10–14 years. Importantly, the data on childhood social stressors were collected prospectively, thus obviating concerns regarding retrospective report.

The follow-back approach used in the present study meant that sexual identity was assessed at a later time point than were BMI and social stressors. We recognize, however, that sexual identity is a fluid developmental process as opposed to an event that occurs at a specific time. Sexual development and attraction are processes that begin earlier than identity development, age of disclosure or coming out, or sexual debut.[34] Maturation of the adrenal axis, at about 5 to 8 years of age, is relevant to sexuality and attachment.[35] Data from studies conducted across cultures provide evidence that sexual attraction and orientation emerge in childhood, suggesting that same-sex attraction emerges several years prior to LGB identity.[36,37] Late adolescents and young adults who identify as lesbian/gay or bisexual report that growing up they always felt “different.”[38] This is an important conceptualization of sexual identity given the models we tested. Typically, in tests of mediation the temporal order is one in which the independent variable is measured prior to the mediator, and the dependent variable following the mediator. In our test of social stress mediators of the association between sexual identity and BMI, we conceptualized LGB status as reflecting the full developmental process leading to sexual identity, including the emergence of same-sex attraction in childhood. Thus, the temporal occurrence of the mediator was not tied to a specific age or date, but instead to the developmental period during which the emergence of identity began.

One interpretation of our data is that girls who experience same sex attraction in childhood become isolated and lonely recognizing the stigma associated with same-sex attraction. Those who engage in pre-sexual behaviors with girls may be ostracized or bullied, again due to stigma and discrimination, as has shown to be the case for older adolescents.[21] Because we did not begin asking about sexual attraction until late adolescence, we are unable to test these hypotheses. Future studies will need to adequately measure the ontogeny of sexual attraction, identity, and gender expression earlier in in life and in a more nuanced manner in order to determine whether and how early social experiences play a primary role in later health disparities for lesbian and bisexual females.

We note several limitations of the present study. First, although BMI is strongly correlated with other indices of adiposity in children and adolescents, such as skin fold thickness and dual x-ray absorptiometry, it is an imperfect measure of adiposity both because it is indirect and is prone to measurement error.[39–41] Second, although BMI did not differ between lesbians and bisexuals, combining populations of individuals into a non-heterosexual group may have obscured differences in predictors of BMI. Moreover, we did not assess gender identity; expression of gendered behavior may also elicit social stress in childhood. Third, we limited the definition of sexual minority to identification as lesbian or bisexual in a single wave of data collection. Sexual identity is not constant for all individuals, and other dimensions of sexuality, such as attraction, may reveal differences among groups. Moreover, socially desirable reporting and or fear of disclosure may have led to underreporting of LGB status. Finally, although our analyses controlled for baseline BMI and race, other unmeasured confounders may have impacted our capacity to validly assess mediation and moderation effects.

In conclusion, the results from the present study extend the existing literature on health disparities for sexual minority females by demonstrating that differences in BMI between adolescents who later identify as lesbian/gay or bisexual, compared to adolescents who identify as heterosexual, emerge relatively early in life, and that social stressors including peer victimization and loneliness may be important social determinants of later health disparities for lesbian/gay and bisexual individuals.

## References

[1] Eliason MJ, Ingraham N, Fogel SC, McElroy JA, Lorvick J, Mauery DR, et al A systematic review of the literature on weight in sexual minority women. *Womens Health Issues* 2015;25:162–75. doi: 10.1016/j.whi.2014.12.001 25747521 10.1016/j.whi.2014.12.001

[2] Dilley JA, Simmons KW, Boysun MJ, Pizacani BA, Stark MJ. Demonstrating the importance and feasibility of including sexual orientation in public health surveys: Health disparities in the Pacific Northwest. *Am J Public Health* 2010;100:460–467. doi: 10.2105/AJPH.2007.130336 19696397 10.2105/AJPH.2007.130336 PMC2820072

[3] Cochran SD, Mays VM. Physical health complaints among lesbians, gay men, and bisexual and homosexually experienced heterosexual individuals: Results from the California Quality of Life Survey. *Amer J Public Health* 2007;97:2048–55. 17463371 10.2105/AJPH.2006.087254 PMC2040376

[4] Diamant AL, Wold C. Sexual orientation and variation in physical and mental health status among women. *J Women Health* 2003;12:41–49. 10.1089/154099903321154130 12639368

[5] Strutz KL, Herring AH, Halpern CT. Health disparities among young adult sexual minorities in the U.S. *Am J Prev Med* 2015;48:76–88. doi: 10.1016/j.amepre.2014.07.038 25241194 10.1016/j.amepre.2014.07.038 PMC4274226

[6] Institute of Medicine. *The Health of Lesbian*, *Gay*, *Bisexual*, *and Transgender People*: *Building a Foundation for Better Understanding* Washington, DC: The National Academies Press; 2011. 22013611

[7] Hagman E, Reinehr T, Kowalski J, Ekbom A, Marcus C, Holl RW. Impaired fasting glucose prevalence in two nationwide cohorts of obese children and adolescents. *Int J Obes* 2014;38:40–5. 10.1038/ijo.2013.124 PMC3884136 23828099

[8] Cappuccio FP, Taggart FM, Kandala NB, Currie A, Peile E, Stranges S, et al Meta-analysis of short sleep duration and obesity in children and adults. *Sleep* 2008;31:619–26. 18517032 10.1093/sleep/31.5.619 PMC2398753

[9] Berenson GS, Srinivasan SR, Bao W, Newman WP 3rd, Tracy RE, Wattigney WA. Association between multiple cardiovascular risk factors and atherosclerosis in children and young adults. The Bogalusa Heart Study. *N Engl J Med* 1998;338:1650–6. doi: 10.1056/NEJM199806043382302 9614255 10.1056/NEJM199806043382302

[10] Katz-Wise SL, Blood EA, Milliren CE, Calzo JP, Richmond TK, Gooding HC, et al Sexual orientation disparities in BMI among U.S. adolescents and young adults in three race/ethnicity groups. *J Obes* 2014;2014:537242 doi: 10.1155/2014/537242 24872890 10.1155/2014/537242 PMC4020453

[11] Austin SB, Ziyadeh NJ, Corliss HL, Haines J, Rockett HR, Wypij D, et al Sexual orientation disparities in weight status in adolescence: findings from a prospective study. *Obesity* 2009;17:776–82. 10.1038/oby.2009.72 PMC2756182 19300430

[12] Hadland SE, Austin SB, Goodenow CS, Calzo JP. Weight misperception and unhealthy weight control behaviors among sexual minorities in the general adolescent population. *J Adolesc Health* 2014;54:296–303. doi: 10.1016/j.jadohealth.2013.08.021 24182939 10.1016/j.jadohealth.2013.08.021 PMC3943999

[13] Braveman P, Egerter S, Williams DR. The social determinants of health: Coming of age. *Annu Rev Public Health* 2011;32:381–98. doi: 10.1146/annurev-publhealth-031210-101218 21091195 10.1146/annurev-publhealth-031210-101218

[14] Matthews KA, Gallo LC. Psychological perspectives on pathways linking socioeconomic status and physical health. Annu Rev Psychol. 2011;62:501–30 doi: 10.1146/annurev.psych.031809.130711 20636127 10.1146/annurev.psych.031809.130711 PMC3121154

[15] Hawkley LC, Cacioppo JT. Loneliness matters: a theoretical and empirical review of consequences and mechanisms. *Ann Behav Med* 2010;40:218–27. doi: 10.1007/s12160-010-9210-8 20652462 10.1007/s12160-010-9210-8 PMC3874845

[16] Heath NM, Chesney SA, Gerhart JI, Goldsmith RE, Luborsky JL, Stevens NR, et al Interpersonal violence, PTSD, and inflammation: potential psychogenic pathways to higher C-reactive protein levels. *Cytokine* 2013;63:172–8. doi: 10.1016/j.cyto.2013.04.030 23701836 10.1016/j.cyto.2013.04.030 PMC3731749

[17] Beatty DL, Matthews KA. Unfair treatment and trait anger in relation to nighttime ambulatory blood pressure in Black American and white adolescents. *Psychosom Med* 2009;71:813–20. doi: 10.1097/PSY.0b013e3181b3b6f8 19661190 10.1097/PSY.0b013e3181b3b6f8 PMC3093296

[18] Skinner ML, Shirtcliff EA, Haggerty KP, Coe CL, Catalano RF. Allostasis model facilitates understanding race differences in the diurnal cortisol rhythm. *Dev Psychopathol* 2011;23:1167–86. doi: 10.1017/S095457941100054X 22018088 10.1017/S095457941100054X PMC3583352

[19] Bogart LM, Elliott MN, Klein DJ, Tortolero SR, Mrug S, Peskin MF, et al Peer victimization in fifth grade and health in tenth grade. *Pediatrics* 2014;133:440–7. doi: 10.1542/peds.2013-3510 24534401 10.1542/peds.2013-3510 PMC4530298

[20] Liu H, Umberson D. Gender, stress in childhood and adulthood, and trajectories of change in body mass. *Soc Sci Med* 2015;139:61–69. doi: 10.1016/j.socscimed.2015.06.026 26151391 10.1016/j.socscimed.2015.06.026 PMC4519422

[21] Johns MM, Lowry R, Demissie Z, Robin L. Harassment and mental distress among adolescent female students by sexual identity and BMI or perceived weight status. *Obesity*;25:1421–1427. doi: 10.1002/oby.21850 28494125 10.1002/oby.21850 PMC5572140

[22] Keenan K, Hipwell AE, Chung T, Stepp SD, Stouthamer-Loeber M, Loeber R, et al The Pittsburgh Girls Studies: Overview and initial findings. *J Clin Child Adolesc Psychol* 2010;39:506–521. doi: 10.1080/15374416.2010.486320 20589562 10.1080/15374416.2010.486320 PMC2946599

[23] Chandra A, Mosher WD, Copen C, Sionean C. Sexual behavior, sexual attraction, and sexual identity in the United States: Data from the 2006–2008 National Survey of Family Growth. 2011. *Natl Health Stat Rep* 2011; 36:1–36. 21560887

[24] Vernberg EM, Jacobs AK, Hershberger SL. Peer victimization and attitudes about violence during early adolescence. *J Clin Child Psychol* 1999;28:386–95. doi: 10.1207/S15374424jccp280311 10446688 10.1207/S15374424jccp280311

[25] Asher SR, Wheeler VA. Children's loneliness: a comparison of rejected and neglected peer status. *J Consult Clin Psychol* 1985;53:500–5. 4031205 10.1037//0022-006x.53.4.500

[26] Pfister R, Schwarz K, Carson R, Jancyzk M. Easy methods for extracting individual regression slopes: Comparing SPSS, R, and Excel. *Tutor Quant Methods Psychol* 2013;9:72–78.

[27] Ogden CL, Carroll MD, Fryar CD, Flegal KM. Prevalence of obesity among adults and youth: United States, 2011–2014. *NCHS Data Brief* 2015;219:1–8. 26633046

[28] Hayes AF. *Introduction to mediation*, *moderation*, *and conditional process analysis* New York, NY: The Guilford Press; 2013.

[29] Preacher KJ, Hayes AF. Asymptotic and resampling strategies for assessing and comparing indirect effects in multiple mediator models. *Behav Res Meth* 2008;40:879–891. 10.3758/brm.40.3.879 18697684

[30] Kuczmarski RJ, Ogden CL, Guo SS, Grummer-Strawn LM, Flegal KM, Mei Z, et al 2000 CDC growth charts for the United States: Methods and development. National Center for Health Statistics. *Vital Health Stats* 2002;11. 12043359

[31] Matthews AK, Hotton A, DuBois S, Fingerhut D, Kuhns LM. Demographic, psychosocial, and contextual correlates of tobacco use in sexual minority women. *Res Nurs Health* 2011;34:141–52. doi: 10.1002/nur.20427 21305575 10.1002/nur.20427

[32] Smith HA, Markovic N, Danielson ME, Matthews A, Youk A, Talbott EO, et al Sexual abuse, sexual orientation, and obesity in women. *J Women’s Health* 2010;19:1525–32. 10.1089/jwh.2009.1763 PMC2941402 20524896

[33] Jun HJ, Corliss HL, Nichols LP, Pazaris MJ, Spiegelman D, Austin SB. Adult body mass index trajectories and sexual orientation: The Nurses' Health Study II. *Am J Prev Med* 2012;42:348–54. doi: 10.1016/j.amepre.2011.11.011 22424247 10.1016/j.amepre.2011.11.011 PMC3309458

[34] Grov C, Bimbi DS, Nanin JE, Parsons JT. Race, ethnicity, gender, and generational factors associated with the coming-out process among lesbian, and bisexual individuals. *J Sex Res* 2006;43:115–21 doi: 10.1080/00224490609552306 16817058 10.1080/00224490609552306

[35] Herdt G, McClintock M. The magical age of 10. *Arch Sex Behav* 2000; 29:587–606 11100264 10.1023/a:1002006521067

[36] Del Guidice M, Belsky J. Sex differences in attachment emerge in middle childhood: An evolutionary hypothesis. *Child Dev Perspec* 2010;4:97–105.

[37] McClelland SI, Rubin JD, Bauermeister JA. "I Liked Girls and I Thought They Were Pretty": Initial Memories of Same-Sex Attraction in Young Lesbian and Bisexual Women. *Arch Sex Behav* 2015; 44:1–15. 25987490 10.1007/s10508-015-0507-3

[38] Herdt G, Boxer *Children of Horizons*, 1993;Beacon Press, Boston.

[39] Laurson KR, Eisenmann JC, Welk GJ. Body Mass Index standards based on agreement with health-related body fat. *Am J Prev Med* 2011;41:S100–5. doi: 10.1016/j.amepre.2011.07.004 21961608 10.1016/j.amepre.2011.07.004

[40] Bartok CJ, Marini ME, Birch LL. High body mass index percentile accurately reflects excess adiposity in white girls. J *Am Diet Assoc* 2011;111:437–41. doi: 10.1016/j.jada.2010.11.015 21338745 10.1016/j.jada.2010.11.015

[41] Himes JM. Challenges of accurately measuring and using BMI and other indicators of obesity in children. *Pediatrics* 2009;124;S3–S22. doi: 10.1542/peds.2008-3586D 19720665 10.1542/peds.2008-3586D

